# EDTA-FISH: A Simple and Effective Approach to Reduce Non-specific Adsorption of Probes in Fluorescence *in situ* Hybridization (FISH) for Environmental Samples

**DOI:** 10.1264/jsme2.ME20062

**Published:** 2020-06-27

**Authors:** Yuki Morono, Kengo Kubota, Daisuke Tsukagoshi, Takeshi Terada

**Affiliations:** 1 Geomicrobiology Group, Kochi Institute for Core Sample Research, Japan Agency for Earth-Marine Science and Technology (JAMSTEC), Monobe B200, Nankoku, Kochi 783–8502, Japan; 2 Department of Civil and Environmental Engineering, Tohoku University, 6–6–06 Aoba, Aramaki, Aoba-ku, Sendai, Miyagi 980–8579 Japan; 3 Marine Works Japan LTD, Oppama-higashi 3–54–1, Yokosuka 237–0063, Japan

**Keywords:** Fluorescence *in situ* hybridization (FISH), marine sediments, EDTA

## Abstract

Fluorescence *in situ* hybridization (FISH) is a widely used molecular technique in microbial ecology. However, the non-specific adsorption of fluorescent probes and resulting high intensity of background signals from mineral particles hampers the specific detection of microbial cells in grain-rich environmental samples, such as subseafloor sediments. We herein demonstrated that a new buffer composition containing EDTA efficiently reduced the adsorption of probes without compromising the properties of the FISH-based probing of microbes. The inclusion of a high concentration of EDTA in the buffer in our protocol provides a simple and effective approach for reducing the background in FISH for environmental samples.

The direct visualization of microbes by fluorescence *in situ* hybridization (FISH) allows for the spatial and temporal identification of the dynamics of individual active microbial populations and is widely used in microbial ecology (Amann and Fuchs, 2008; Dianou *et al.*, 2012; Hirakata *et al.*, 2015). Recent developments that have increased the sensitivity of FISH (Furukawa *et al.*, 2006; Kawakami *et al.*, 2010; Kubota, 2013) as well as its combination with techniques such as transmission electron microscopy (Halary *et al.*, 2011) and nano-scale secondary ion mass spectrometry (Dekas and Orphan, 2011; Morono *et al.*, 2011) have further enhanced its utility in identifying specific microbes with respective functions in an environment.

However, the application of FISH-based probing is hampered by the presence of inorganic mineral grains that adsorb DNA (Ogram *et al.*, 1988; Bezanilla *et al.*, 1995; Saeki *et al.*, 2010). When a standard FISH protocol is applied to subseafloor sediment samples, bright fluorescence is frequently observed on mineral particles, speculatively due to the adsorption of fluorescent oligonucleotide probes onto mineral surfaces. Furthermore, the DNA-binding dyes used to stain all microbes in samples are adsorbed onto mineral surfaces. This non-specific adsorption negatively affects the identification of active microbial populations.

To prevent the non-specific adsorption of fluorescent probes and, thus, reduce background signals, we used a high concentration of EDTA in the hybridization buffer for FISH. EDTA is a chelating agent that is widely used to chelate divalent cations, thereby preventing the degradation of DNA by deoxyribonucleases. A previous study demonstrated that the presence of a high concentration of EDTA increased the efficiency of DNA recovery in extractions from environmental samples by preventing DNA adsorption by mineral particles in soil (Krsek and Wellington, 1999; Rai *et al.*, 2010). If DNA adsorption is prevented by a high concentration of EDTA, the non-specific adsorption of oligonucleotide probes for FISH may also be avoided. Therefore, we examined the effectiveness of EDTA to reduce background signals caused by the non-specific binding of fluorescent oligonucleotide probes during hybridization.

Three different buffer compositions were investigated: a standard FISH buffer composition (0.9 M NaCl, 20‍ ‍mM Tris/HCl, 0.01% SDS, and 5–35% formamide) and buffers containing 142 or 250‍ ‍mM EDTA instead of 0.9 M NaCl (designated as EDTA-FISH). An EDTA solution (pH 8.0) (generated using EDTA·2Na and NaOH for pH adjustments) contained approximately 750‍ ‍mmols of Na^+^ for 250‍ ‍mmol of EDTA ions. Since Na^+^ is the ion that affects the properties of hybridization, NaCl was omitted to avoid high Na^+^ concentrations in the hybridization buffer for EDTA-FISH. The marine subsurface sediment samples used in the present study were representative of typical organic-rich deep (sediment depth of 219 m) and ancient (*ca.* 460,000 years) sedimentary habitats in the northwestern Pacific Ocean off the Shimokita Peninsula of Japan, as described previously (Morono *et al.*, 2011). Microbial activity was stimulated by the addition of methane to the headspace of the test tube and incubated at 10°C for 2 months. Samples were fixed with paraformaldehyde, washed with PBS, and preserved in EtOH/PBS (1:1) at –20°C until used. Sediment slurry was placed on a glass slide, embedded in low gelling point agarose (concentration 0.1%; MetaPhor, Lonza), dehydrated in an ethanol series (50, 80, and 99.5% ethanol), and then air-dried. A mixture of the EUB338 I, II, and III probes (Daims *et al.*, 1999) labeled with Alexa Fluor 488 was used. Formamide concentrations ranged between 5 and 35%, and incubation temperatures and durations for the hybridization and washing steps were 46°C for 2 h and 48°C for 20‍ ‍min, respectively. The NON338 probe (complimentary to EUB338 I) was used as a negative control. As shown in Fig. 1, fluorescence signals were obtained in hybridizations with both the EUB338 and NON338 probes after standard FISH, which clearly demonstrated that the non-specific adsorption of probes compromised the specific detection of microbes. On the other hand, EDTA-FISH effectively reduced the non-specific adsorption of the NON338 probe, while the intensity of signals from microbial cells was unchanged in the hybridization with the EUB338 probe. In EDTA-FISH, we used two different concentrations of EDTA (142 and 250‍ ‍mM) to assess whether EDTA concentrations affect the adsorption of probes to sediment particles. Although the images shown in Fig. 1 did not appear to differ, a slightly lower background signal was observed with 250‍ ‍mM EDTA by eye-based observations; therefore, 250‍ ‍mM EDTA was subsequently used.

To evaluate the effects of EDTA on the hybridization properties of probes and specificity of FISH, we conducted experiments to investigate probe dissociation and mismatch discrimination. Dissociation curves for EDTA-FISH and standard FISH were investigated at various concentrations of formamide in both the hybridization and washing buffers. Hybridization and washing times were 3 h and 15‍ ‍min, respectively. The EUB338 and GAM42a probes were evaluated using *Escherichia coli* cells. Fluorescent images were obtained under a fixed exposure time, and signal intensities were measured using daime software (Daims *et al.*, 2006). The dissociation curves obtained by EDTA-FISH shifted to a lower formamide concentration, and the degrees of these shifts differed (Fig. 2a and b). Only a slight difference was observed for the GAM42a probe, whereas the EUB338 probe showed a marked change (approximately 10% formamide difference). Differences in Na^+^ concentrations may partially explain the shift observed in the optimum formamide concentration. The concentration of Na^+^ in hybridization buffer was approximately 0.75 M when 250‍ ‍mM EDTA was used (EDTA·2Na and NaOH were used for pH adjustments), which was 0.15 M lower than that in standard FISH hybridization buffer. Theoretically, the decrease of 0.15 M in Na^+^ reduced the melting temperature of oligonucleotides by 1.3°C (=16.6 log [0.9/0.75], [Wilkinson, 1992]), which corresponded to approximately 3% of formamide (0.5°C/% formamide for a DNA-RNA hybrid [Wilkinson, 1992]). The present results clearly demonstrated that an optimum formamide concentration needs to be selected for EDTA-FISH probe-by-probe. Mismatch experiments, for which we selected two oligonucleotide probes, GAM42a (Manz *et al.*, 1992) and SRB385 (Amann *et al.*, 1990), were conducted to assess the effects of EDTA on mismatch discrimination. The GAM42a probe was used to evaluate single mismatch discrimination using paraformaldehyde-fixed *E. coli* (*Gammaproteobacteria*) and *Comamonas testosteroni* (*Betaproteobacteria*) pure cultures. The SRB385 probe was for two mismatch discrimination, and fixed pure cultures of *Desulfovibrio vulgaris* and *Deinococcus radiodurans* were used. The results shown in Fig. 3 demonstrated that EDTA-FISH distinguished two mismatches (Fig. 3c), whereas the discrimination of a single mismatch was not possible (Fig. 3a). The use of a competitor probe was necessary for single mismatch discrimination (Fig. 3b). These results are consistent with standard FISH (Manz *et al.*, 1992). Thus, no apparent effect of using EDTA on the selectivity of FISH was observed. However, it is important to note that an optimum formamide concentration needs to be selected for each probe. Differences in Na^+^ concentrations between the standard FISH and EDTA-FISH hybridization buffers caused a shift in the concentration of formamide, which varied depending on the probes. Although the mechanisms responsible for differences in the degree of the formamide concentration shift have not yet been elucidated and are out of the scope of the present study, a reduction in the background adsorption of oligonucleotide probes was achieved without compromising any critical property of FISH probing.

In the present study, we examined the utility of EDTA for reducing the background signals of FISH caused by the non-specific adsorption of fluorescent oligonucleotide probes. The replacement of 0.9 M NaCl with 250‍ ‍mM EDTA in the hybridization buffer effectively reduced the non-specific adsorption of probes, while the hybridization of probes to target molecules in microbial cells was maintained. Although slight differences were observed in hybridization characteristics and the optimization of hybridization conditions needs to be conducted probe-by-probe, the capacity for mismatch discrimination was unchanged in EDTA-FISH. This simple EDTA-FISH procedure has potential for expansion to reducing background signals in a more advanced FISH protocol, such as hybridization chain reaction-FISH (Yamaguchi *et al.*, 2015), or other sensitized protocols of FISH in the future.

## Citation

Morono, Y., Kubota, K., Tsukagoshi, D., and Terada, T. (2020) EDTA-FISH: A Simple and Effective Approach to Reduce Non-specific Adsorption of Probes in Fluorescence *in situ* Hybridization (FISH) for Environmental Samples. *Microbes Environ ***35**: ME20062.

https://doi.org/10.1264/jsme2.ME20062

## Figures and Tables

**Fig. 1.**
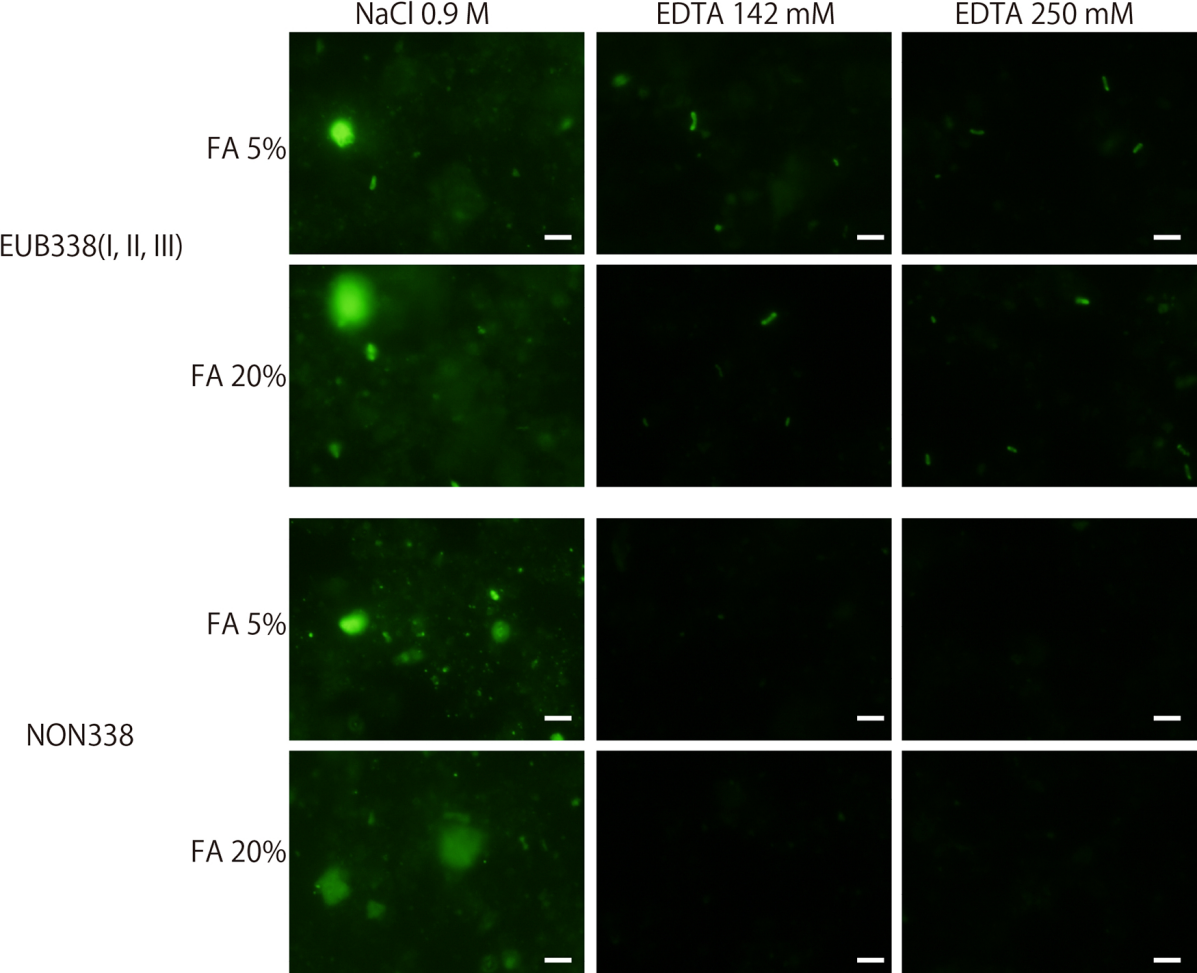
FISH trials with different buffer compositions and probes. In the standard FISH protocol, 0.9 M NaCl was used. EDTA (142 and 250‍ ‍mM) was used instead of NaCl in the hybridization buffer for EDTA-FISH. EUB338 (a mixture of I, II, and III) and NON338 probes were used to assess the degree of the non-specific adsorption of probes onto non-cell sedimentary particles in samples. Bars are 5‍ ‍μm.

**Fig. 2.**
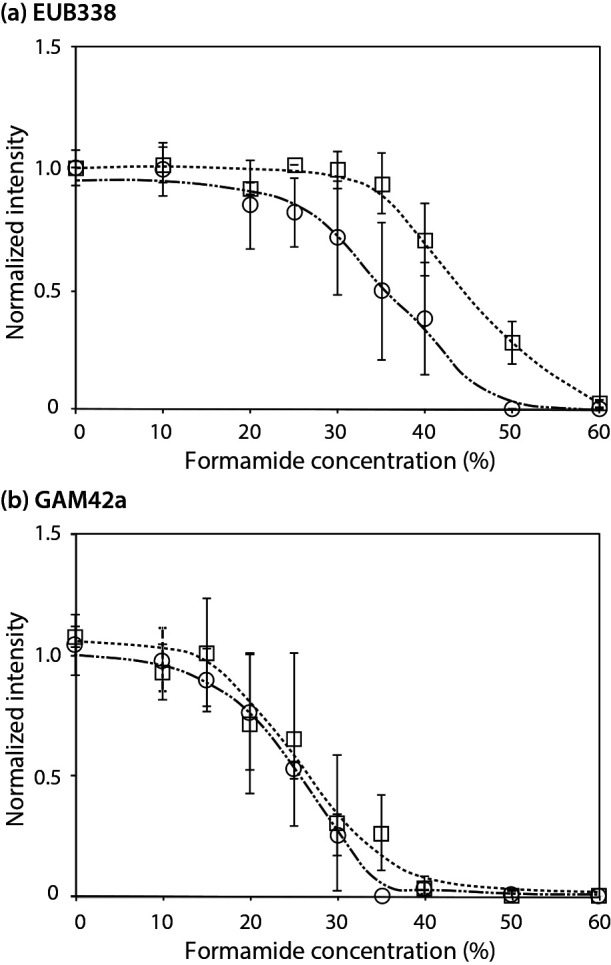
Dissociation curves of EUB338 (a) and GAM42a (b) obtained by standard FISH (□) and EDTA-FISH (○). The signal intensity of EUB338 at 0% formamide was set as 1.0 for the normalization of both dissociation curves.

**Fig. 3.**
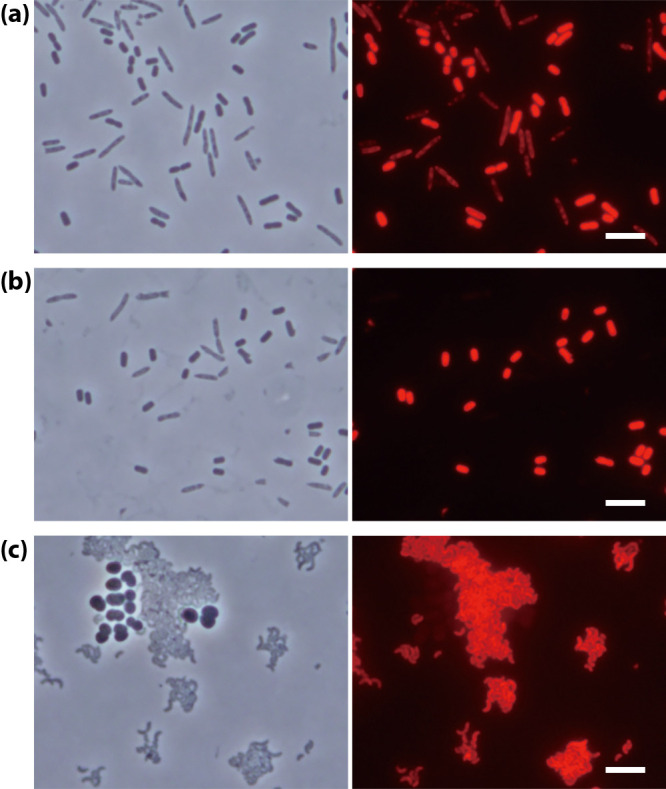
Evaluation of the mismatch discrimination capacity of EDTA-FISH. Phase contrast and epifluorescent micrographs show identical fields. Artificial mixtures of *Escherichia coli* (short rod) and *Comamonas testosteroni* (rod) (a and b) and *Desulfovibrio vulgaris* (curved rod) and *Deinococcus radiodurans* (coccus) (c) were hybridized with the GAM42a probe (a), the GAM42a probe with a competitor probe (b) and the SRB385 probe (c). Bars are 5‍ ‍μm.
